# Risks for Central Nervous System Diseases among Mobile Phone Subscribers: A Danish Retrospective Cohort Study

**DOI:** 10.1371/journal.pone.0004389

**Published:** 2009-02-05

**Authors:** Joachim Schüz, Gunhild Waldemar, Jørgen H. Olsen, Christoffer Johansen

**Affiliations:** 1 Institute of Cancer Epidemiology, Danish Cancer Society, Copenhagen, Denmark; 2 Memory Disorder Research Group, Department of Neurology, Copenhagen University Hospital, Copenhagen, Denmark; University of Louisville, United States of America

## Abstract

The aim of this study was to investigate a possible link between cellular telephone use and risks for various diseases of the central nervous system (CNS). We conducted a large nationwide cohort study of 420 095 persons whose first cellular telephone subscription was between 1982 and 1995, who were followed through 2003 for hospital contacts for a diagnosis of a CNS disorder. Standardized hospitalization ratios (SHRs) were derived by dividing the number of hospital contacts in the cohort by the number expected in the Danish population. The SHRs were increased by 10–20% for migraine and vertigo. No associations were seen for amyotrophic lateral sclerosis, multiple sclerosis or epilepsy in women. SHRs decreased by 30–40% were observed for dementia (Alzheimer disease, vascular and other dementia), Parkinson disease and epilepsy among men. In analyses restricted to subscribers of 10 years or more, the SHRs remained similarly increased for migraine and vertigo and similarly decreased for Alzheimer disease and other dementia and epilepsy (in men); the other SHRs were close to unity. In conclusion, the excesses of migraine and vertigo observed in this first study on cellular telephones and CNS disease deserve further attention. An interplay of a healthy cohort effect and reversed causation bias due to prodromal symptoms impedes detection of a possible association with dementia and Parkinson disease. Identification of the factors that result in a healthy cohort might be of interest for elucidation of the etiology of these diseases.

## Introduction

The worldwide spread of the use of mobile phones has raised concern about possible adverse health effects [1], [2]. Most of the epidemiological studies conducted so far have addressed the risk for brain tumours. There is accruing evidence that mobile phone use does not increase the risk for these tumours among short-term users, but the picture is less clear for frequent users for 10 years or more: whereas a substantial increase in risk is unlikely, a small-to-moderate increase cannot be ruled out [3]–[7]. During operation of a mobile phone, the antenna emits radiofrequency electromagnetic fields (RF-EMF), which can penetrate 4–6 cm into the human brain [8], [9], leading to relatively localized exposure. Hence, brain tumours are indeed a major outcome of interest, but for the same reason it would be important to investigate the risks for other diseases of the central nervous system (CNS). Recent reviews of epidemiological studies of associations between exposure to extremely low-frequency electromagnetic fields (ELF-EMF) in certain occupations and the risk for CNS disease indicated increased risks for Alzheimer disease and amyotrophic lateral sclerosis (ALS) but not for vascular dementia, epilepsy or Parkinson disease [10]–[12]. Despite the fact that the emission from mobile phones is a combination of RF-EMF and pulsed ELF-EMF [13], no studies have investigated the association between use of mobile phones and risks for CNS disease.

In this registry-based study, we used a retrospective cohort of Danish mobile phone subscribers between 1982 and 1995 to compare their hospitalisation rates for CNS diseases with those of the general population for the same diseases. To our knowledge, this is the first study in which possible associations between mobile phone use and the occurrence of migraine, vertigo and more severe CNS diseases, such as Alzheimer disease, vascular dementia, Parkinson disease, ALS, multiple sclerosis and epilepsy, have been examined systematically.

## Methods

The composition of the cohort has been described previously [3]. In brief, we obtained the records of all 723 421 mobile phone subscriptions in Denmark during the period 1982–1995. We deleted 200 507 corporate subscriptions, as the individual users could not be identified. A further 102 819 records were excluded because of: duplicate addresses (one of them removed), errors in name or address, a non-residential address, subscriber under 18 years of age at first subscription, subscriber a permanent resident of Greenland or the Faroe Islands, or the subscriber asked to be excluded from the study (*n* = 53). The final study cohort comprised 420 095 private mobile phone subscribers.

We used the date of the first subscription as the entry date into the cohort, but persons remained in the cohort even when they stopped the respective subscription. We have done this for two reasons. First, years since first mobile phone use were preferred to the number of years of mobile phone use to estimate exposure in most of the cancer-related mobile phone studies, too, and, if both metrices were used, the results differed only marginally (e.g. [4]–[7]); an effect most likely attributable to the habit that the amount of mobile phone use varies over time but persons who once started using mobile phones rarely completely stop using them. Second, starting with a new subscription requires a new identification of the individual user and failure of identication of a former mobile phone subscriber would be either an individual who has indeed stopped using a mobile phone or an individual who remained to be a subscriber but was not identified as such by us.

Through record linkage of cohort members by name and address with the Central Population Register, their personal identification numbers were obtained, with data on vital status and date of death or emigration. Using the personal identification number, we linked the cohort members to the files of the Hospital Discharge Registry in order to ascertain hospitalisations for CNS diseases. This Registry contains information on all hospitalisations in Denmark since 1977, and, from 1994 onwards, information on outpatients. Cohort members were followed until their first hospital contact (hospitalisations up to 1994 and hospital contacts thereafter) for each disease. Follow-up began on the date of first subscription and ended on the date of first hospital contact, date of death, date of emigration or 31 December 2003, whichever came first. The following groups of CNS diseases were defined: Alzheimer disease (ICD-10 F00.0,.1,.2,.9; G30.0,.1,.8,.9), vascular dementia (ICD-10 F01.0,.1,.2,.3,.8,.9), other dementia (including other specific disorders with dementia, unclassified dementia and other unspecified degenerative CNS diseases; ICD-10 A81.0; B22.0; F02.0,.1,.2,.3,.4,.8; F03.9; G10; G20 except G20.9; G31.0,.1,.8,.9), Parkinson disease (ICD-10 G20.9), ALS (ICD-10 G12.2,.8,.9), multiple sclerosis (ICD-10 G35.9), and epilepsy (ICD-10 G40; G41). Furthermore, we obtained information on hospitalisations for migraine (ICD-10 G43) and vertigo (ICD-10 A88.1; H81.1; H81.3). All the diagnoses, including the action diagnosis and up to 20 additional recorded diagnoses, were used as indicators of the outcome. All the outcomes were measured after hospital discharge requiring at least 24 h of admission or outpatient visits to the hospital. This registry-based study was approved by the Danish Ethical Committee System (KF 01-075/96), the Danish Data Protection Board (1996-1200-121), and the Danish Ministry of Justice (Jnr. 1996-760-0219) and did not require written informed consent by participants. The cellular telephone operators informed their subscribers about the study via their newsletters and the possibility to demand exclusion was provided to everyone, but only 53 subscribers (see above) refused to be included.

The numbers of hospital contacts observed were compared with those expected, which were calculated by multiplying the number of person–years of cohort members by the overall and disease-specific hospital contact rates for primary CNS disease among men and women in the general population of Denmark, in 5-year age groups and calendar periods of observation. In order to exclude cohort members from the reference population, the number of cases of each CNS disease and the respective person–years observed in the cohort were subtracted from the corresponding figures for the total Danish population, and a new set of hospital contact rates was created. Standardized hospitalisation ratios (SHRs) for the various CNS diseases (including migraine and vertigo) and 95% confidence intervals (CIs) were calculated on the assumption of a Poisson distribution of the observed diseases [14]. The SHRs were calculated by gender and for both genders combined, but because of the small number of cases of most CNS diseases in the cohort or lack of difference in the gender-specific effect estimates, only the SHRs for both genders combined are presented in the tables; the only exception is epilepsy, because the gender-specific effect estimates differed. The SHRs are also given by latency, which was calculated as years between the time of first subscription to a mobile phone and the first hospital contact for the respective outcome.

As described earlier [3], we obtained additional data from Statistics Denmark in order to compare the age- and gender-specific average incomes of our cohort members with the respective figures in the general population of Denmark.

## Results

**Figure 1:**
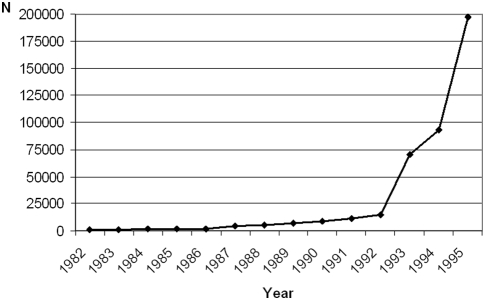
Year of first subscription to a cellular telephone for the 420 095 members of the Danish retrospective cohort of cellular telephone subscribers between 1982 and 1995.

Overall, the 420 095 cohort members represented almost 4 million person–years at risk. As mobile phone use became popular only in the mid-1990s, only 10·6% of all cohort members obtained their first subscription before 1992 (Figure 1). Most subscriptions (46·9%) were taken out in 1995. Before 1992, a large majority of subscribers were in the intermediate age group (30–59 years; 80%), rather than younger (18–29 years, 16%) or older groups (≥60 years, 4%). In 1995, the respective proportions were 62%, 31% and 7%. Women represented 62 542 (15%) of the cohort members.

There was an overall small but statistically significant excess of hospital contacts for migraine and vertigo among cohort members (Table 1). The excess was smallest for subscribers of ≥10 years, but the differences in effect estimates across the four latency groups were generally small. Although the age-adjusted hospital contact rates for migraine or vertigo were higher among women (more than three times for migraine and about 2·5 times for vertigo, a gender ratio similar to that observed in the general population), the absolute numbers of affected women were much smaller because there were fewer women in the subscriber cohort. The effect estimates were, however, similar, being 1·2 for both men and women for migraine and 1·1 for men and 1·2 for women for vertigo.

**Table 1:** Standardized hospitalisation ratios (SHRs) and 95% confidence intervals (CIs) for migraine and vertigo among 420 095 subscribers to cellular telephones in Denmark, 1982–1995, followed up through 31 December 2003.

Condition	Latency (years)*	Observed	Expected	SHR	CI
Migraine	1	148	117.0	1.3	1.1–1.5
	1–4	611	503.6	1.2	1.2–1.3
	5–9	586	500.3	1.2	1.1–1.3
	≥10	56	53.3	1.1	0.8–1.4
	Total	1401	1174.2	1.2	1.1–1.3
Vertigo	1	137	126.3	1.1	0.9–1.3
	1–4	750	658.0	1.1	1.1–1.2
	5–9	1148	1023.2	1.1	1.1–1.2
	≥10	191	187.1	1.0	0.9–1.2
	Total	2226	1994.6	1.1	1.1–1.2

* Time since first subscription to a cellular telephone.

The SHRs for Alzheimer disease, vascular dementia and other dementia were all statistically significantly decreased (Table 2). A significantly decreased SHR was also observed for Parkinson disease, whereas the SHRs for ALS and multiple sclerosis were close to unity. For epilepsy, there was a significantly decreased SHR among men but not women, for which the SHR was slightly above 1. Subscribers for ≥10 years or more were of particular interest because of the longer latency, but the SHRs for this group were all close to or below unity. Statistically significantly reduced SHRs were seen for Alzheimer disease and for epilepsy among males.

**Table 2:** Standardized hospitalisation ratios (SHRs) and 95% confidence intervals (CIs) for diseases of the central nervous system among 420 095 subscribers to cellular telephones in Denmark, 1982–1995, followed up through 31 December 2003.

Disease	Latency (years)*	Observed	Expected	SHR	CI
Alzheimer disease	1	1	5.4	0.2	0.0–1.0
	1–4	25	33.1	0.8	0.5–1.1
	5–9	50	63.4	0.8	0.6–1.0
	≥10	5	13.1	0.4	0.1–0.9
	Total	81	114.9	0.7	0.6–0.9
Vascular dementia	1	2	4.3	0.5	0.1–1.7
	1–4	19	26.8	0.7	0.4–1.1
	5–9	34	52.9	0.6	0.4–0.9
	≥10	13	11.8	1.1	0.6–1.9
	Total	68	95.7	0.7	0.5–0.9
Other dementia	1	21	40.1	0.5	0.3–0.8
	1–4	131	198.7	0.7	0.5–0.8
	5–9	198	257.5	0.8	0.7–0.9
	≥10	33	54.8	0.6	0.4–0.9
	Total	383	551.1	0.7	0.6–0.8
Parkinson disease	1	10	21.4	0.5	0.2–0.9
	1–4	82	106.0	0.8	0.6–1.0
	5–9	110	136.1	0.8	0.7–1.0
	≥10	35	31.6	1.1	0.8–1.5
	Total	237	295.1	0.8	0.7–0.9
Amyotrophic lateral sclerosis	1	11	8.1	1.4	0.7–2.4
	1–4	42	37.2	1.1	0.8–1.5
	5–9	44	44.5	1.0	0.7–1.3
	≥10	7	10.1	0.7	0.3–1.4
	Total	104	99.9	1.0	0.9–1.3
Multiple sclerosis	1	61	51.3	1.2	0.9–1.5
	1–4	222	217.4	1.0	0.9–1.2
	5–9	220	211.1	1.0	0.9–1.2
	≥10	25	29.4	0.9	0.6–1.3
	Total	528	509.3	1.0	0.9–1.1
Epilepsy (men)	1	201	249.9	0.8	0.7–0.9
	1–4	752	1015.4	0.7	0.7–0.8
	5–9	716	979.4	0.7	0.7–0.8
	≥10	98	176.0	0.6	0.5–0.7
	Total	1767	2420.6	0.7	0.7–0.7
Epilepsy (women)	1	41	37.1	1.1	0.8–1.5
	1–4	156	145.2	1.1	0.9–1.3
	5–9	135	129.0	1.0	0.9–1.2
	≥10	5	7.1	0.7	0.2–1.6
	Total	337	318.4	1.1	0.9–1.2

* Time since first subscription to a cellular telephone.

## Discussion

### Summary of Findings

In this cohort study of mobile phone subscribers between 1982 and 1995, we observed weak positive associations with migraine and vertigo but inverse associations with Alzheimer disease, vascular and other dementia, Parkinson disease and epilepsy among men. No associations were seen with ALS, multiple sclerosis or epilepsy among women. These findings were also made for long-term subscribers, i.e. persons who had had a subscription to a mobile phone for 10 or more years before their disease was registered.

Headaches and dizziness are frequent self-reported symptoms associated with mobile phone use [15]–[18], and the evidence that these complaints are causally linked to exposure to RF-EMF is weak [1], [2]. This assessment is based mainly on the absence of consistent effects in human provocation and sleep studies and on the lack of a biologically plausible explanation, although participants in a recent Swedish human provocation study reported headache and vertigo more often after mobile phone use than after sham exposure [19].

Our finding of a higher rate of hospital contacts for migraine among mobile phone subscribers raises the question of what factors lead to a hospital contact, as only a small proportion of migraine patients are referred to hospital [20]. Although high work load has been associated with migraine [21] and work-related stress was more common in frequent mobile phone users in a Swedish–Norwegian cross-sectional survey [18], we had no reason to expect a higher initial rate of migraine in our cohort or a greater likelihood of hospital contacts. The possibility that cohort members with a higher average income than the general population [3], working predominantly in occupations in which mobile telecommunication is an advantage, prefer to go directly to a specialized center in a hospital than to a general practitioner is speculative. Our finding that the excess of migraine was more frequent after the shortest latency might also indicate heightened awareness among mobile phone users. There was, however, no clear pattern of increasing risk for migraine or vertigo with time between exposure and diagnosis of these outcomes.

We found reduced risks for a hospital contact for all types of dementia, including Alzheimer disease, and for Parkinson disease. As there is no biological evidence of a protective effect of mobile phones [1], alternative explanations are needed. One alternative is that the prodromal symptoms of these diseases reduce the likelihood of becoming a mobile phone user. This is in line with the findings for vascular dementia and particularly for Parkinson disease, for which the inverse association was stronger with shorter latency. For Alzheimer disease and other dementia, the picture is less clear, as an inverse association was also seen for persons who had a subscription ≥10 years before hospitalisation. Moreover, the age distribution of our cohort suggests that the start of subscription for most cohort members would have been before the typical age of onset of dementia. Differences in hospitalisation patterns between cohort members and the general population is another possibility; however, as Denmark ensures tax paid, free, equal access to medical health care, this is unlikely.

Our cohort differed in many social and lifestyle aspects from the general population [3], with a higher average income, which is related to a healthier lifestyle. The members were theoretically at lower risk for dementia, as the risk for dementia appears to decrease with a healthy diet [22]–[24], among nonsmokers [25], and with participation in mental, physical and social activities [22]–[26], all of which might be assumed to be more common in our cohort than in the general population. This alone, however, might not explain the 30% decrease in risk, as income is only a proxy for a healthy lifestyle. Furthermore, while the income difference between our cohort and the general population was restricted to men, the decrease in risk was also observed among women. Therefore, it will be important to investigate further the characteristics of our cohort members, such as occupation and lifestyle, in order to identify new hypotheses for the potential risk factors for dementia.

Epilepsy in our study comprised a mixture of idiopathic and symptomatic epilepsies and both prevalent and incident epilepsy, as many cases are diagnosed at an early age, before persons subscribe to a mobile phone [27]. The rate of hospital contacts for epilepsy was decreased among men, perhaps again reflecting a healthy cohort effect or less frequent participation in control examinations by ‘busy’ mobile phone users. There is no straightforward explanation for the restriction of the inverse association to men, but, as discussed below, a healthy cohort effect can be expected to be stronger among men. Furthermore, in Denmark, epileptic subtypes, and therefore susceptibility to their development, differ between genders [28].

We found no association between mobile phones use and ALS and multiple sclerosis. ALS was of particular interest because previous epidemiological studies suggested an association with occupational exposure to power-frequency EMF [10]. The negative finding of our study does not weaken the earlier observations, as the nature of the exposure is quite different; however, it provides a first indication that RF-EMF are not a strong risk factor for ALS.

### Strengths and limitations

Our approach has several strengths. First, the nationwide coverage of subscribers to all Danish mobile phone operators at that time enabled us to achieve the best possible representativity. Secondly, the large size of the cohort and the long follow-up period resulted in effect estimates with narrow confidence intervals. By eliminating exposed cohort members, i.e. mobile phone subscribers, from the comparison population in the calculations of SHRs, we removed a potential source of underestimation of an association. Additionally, we were able to address possible associations many years after first mobile phone subscription. Further strengths of our study are the use of the national Hospital Discharge Registry, which enabled us to investigate morbidity instead of mortality. This Registry is an administrative database which collects data independently of our study hypotheses, providing unbiased data on our outcomes of interest. Outpatients, however, were recorded only from 1994 onwards. We were able to obtain additional data on income from Statistics Denmark and self-reported mobile phone use in a smaller sub-cohort, which assisted us in interpreting our findings. Furthermore the use of an objective measure of exposure, years of subscription, derived from the files of all Danish network providers, increased the possibility of avoiding several methodological limitations inherent in self-reported information on mobile phone use [29].

The study also has limitations. First, use of subscription information raises the possibility of exposure misclassification. Users of mobile phones whose subscriptions are not listed under their names were classified as unexposed in this study and were included in the general population rates used to compute expected values. Furthermore, subscribers who did not actually use a mobile phone were classified as exposed. The misclassification deriving from use of subscriber data is, however, non-differential, so that the expected direction of any bias would be toward underestimation of a true risk. In a previous validation study of our cohort [30], we evaluated the potential for bias by comparing our subscriber list with self-reported information from 822 Danes participating as controls in a case–control study of the possible association between brain tumour risk and use of mobile phones [31]. More than 60% of controls identified in the subscriber cohort characterized themselves as making or receiving calls at least once a week, while the comparison population of non-subscribers included only 16% of such users; hence, the subscriber cohort contains about four times as many regular mobile phone users as the comparison population of non-subscribers. We do not know, however, whether this held true for patients with the outcomes studied in the present analyses.

A second potential limitation of our study is the exclusion of users whose subscription was in the name of their company. This might not only have reduced the proportion of users but perhaps excluded some of the most active ones. We also had no information on new subscribers after 1995, who were therefore included in the reference population. Thus, most of our reference population consists of recent mobile phone users, which could result in underestimation of any association. It is therefore particularly important to subdivide the cohort by latency, as subscribers for ≥10 years were found only in the cohort members.

A third potential limitation is our finding that the persons who subscribed to mobile phones between 1982 and 1995 had a higher income than the general population, opening the possibility of a healthy cohort effect [3]. This is a particular concern for diseases related to a healthy lifestyle, such as less smoking and a healthy diet, and also suggests more frequent contact with hospitals for neurological symptoms. We found that our cohort had reduced incidence rates of lung cancer and many other lifestyle-associated cancers among men [3]. These limitations must be kept in mind in interpreting our findings.

Measuring migraine and vertigo by hospital contacts also has limitations. Vertigo in particular is a broad diagnostic group, used also for dizziness or similar symptoms of an unknown CNS disorder, and can be due to vestibular or cardiac anomalies. While for some patients vertigo is an early symptom of a CNS disorder identified later, for others it is only a short symptomatic episode. Nevertheless, a true association between mobile phone use and vertigo remains a concern, as having an attack in situations such as driving a car or bicylce could have serious consequences.

### Conclusions

It is important to start studying other diseases than brain tumours in relation to mobile phone use, as there are no strong reasons to focus on brain tumours as the main outcome. Our study is the first to investigate a possible association between use of mobile phones and risk of CNS disorders. Most of the observed associations are probably not related to the RF-EMF exposure from mobile phones. Since the study includes the entire population of Denmark and there is no lost to follow-up, the results reflect causality, confounding or an unequal distribution of determinants for being hospitalised like comorbidity. Healthcare behaviour may also be related to mobile phone use affecting especially the results for diseases that only occasionally lead to hospitalisation.

With regard to dementia, an interplay of a healthy cohort effect and reversed causation bias due to prodromal symptoms impedes detection of a possible association, although identification of the factors that contribute to the healthy cohort would be of interest for determining the aetiology of dementia. As interviewed-based case–control studies such as those used to investigate mobile phone use and cancer risk [1] are not an option for dementia, the only study type for addressing this research question would be a prospective cohort study. We observed no association between mobile phone use and ALS, which is reassuring, as this disease has been reported to be associated with occupational exposure to ELF-EMF [10], [11].

The weak but statistically significant associations between mobile phone use and migraine and vertigo deserve further attention. Owing to the high prevalences of these conditions, our observed 10–20% excess of hospitalisations, which reflect only a small proportion of the occurrence of these syndromes, is related to the large absolute numbers of affected persons in our cohort. This would represent a serious public health problem if the associations are confirmed. As a higher risk for car accidents is the only scientifically established adverse consequence of mobile phone use [32], the roles of vertigo, dizziness and headache associated with mobile phone use should be further examined. This study endorses public health recommendations for prudent use of mobile phones, including using wired hand-free sets or other exposure-reducing measures, until more evidence about the possible health effects has been obtained.
